# Empowering motivation: the journey of wheelchair basketball athletes to overcome constraints

**DOI:** 10.3389/fpsyg.2024.1330971

**Published:** 2024-01-29

**Authors:** Halil Sarol

**Affiliations:** Gazi University, Ankara, Türkiye

**Keywords:** wheelchair basketball, athlete, self-determination, motivation, constraints

## Abstract

**Introduction:**

The aim of this phenomenological study is to determine the motivational factors in the participation of wheelchair basketball players in sports according to Self-Determination Theory.

**Methods:**

The study group of the research was determined by the criterion sampling method, which is one of the purposeful sampling methods. Accordingly, thirteen (13) wheelchair athletes constituted the study group. In the research, a personal information form and a semi-structured interview form were prepared as data collection tools within the framework of the self-determination theory. Interviews were conducted face-to-face with the prepared interview forms. The data were analyzed by reflexive thematic analysis method.

**Results:**

Six (6) themes emerged: constraints, coping strategies, appreciation, positive feedback, need for existence, and development. Sixteen sub-themes related to these themes were identified.

**Conclusion:**

The most significant factors causing a lack of motivation in wheelchair basketball athletes were observed to be structural constraints such as access to materials and facilities. Extrinsic sources of motivation were identified as the positive attitudes and behaviors of individuals in their environment, often related to admiration, appreciation, and being set as an example, and this situation has a positive impact on wheelchair basketball athletes. On the other hand, it can be stated that intrinsic motivations such as identity change, gaining self-confidence, and the feeling of success are crucial in overcoming constraints through sports participation.

## 1 Introduction

Sir Ludwig Guttmann introduced sports as a form of rehabilitation that helped increase the functionality and independence of individuals with disabilities after World War II. Since then, it has been recognized that regular participation in sports activities has many psychological, social and physical benefits for people with disabilities (Stephens et al., 2012). Sports play a role in preventing the exclusion of individuals with disabilities from participating in activities, just like their peers without disabilities, and have an impact on their psychological well-being (Moss et al., 2020; Koçak et al., 2023). Sports also provide individuals with disabilities the opportunity to socialize and play a significant role in accepting their disability status (Porretta, 2016). In addition, athletes with disabilities, like athletes without disabilities, are provided with the opportunity to compete with their peers (Steadward, 1990), and like all athletes, they can have some common participation features such as the excitement of competition and being part of the team (Martin, 2019).

Excitement of competition and being a part of a team can be said to be important for every individual. In order to experience these emotions, wheelchair basketball is considered suitable for individuals with disabilities (Snyder et al., 2022). In recent years, there has been a positive increase in the perspective and participation in wheelchairs. In this context, examining the current position of wheelchair basketball for individuals/athletes with disabilities is crucial. In this context, wheelchair basketball and participation in this sport constitute an important subject of examination. The research conducted by Moss et al. (2020) evaluates wheelchair basketball as a socialization opportunity. On the other hand, it is seen to be an important factor in encouraging participation in terms of physical health. In this direction, Ramsden et al. (2023) stated that wheelchair basketball athletes’ physical health and expectations are an important source of motivation. Additionally, Fiorilli et al. (2013) reported that athletes who participated in wheelchair basketball had better psychological well-being than those who did not participate. Moss et al. (2020) see wheelchair basketball as an important element in accepting disability as a ‘meaningful pursuit that opens doors’. Again, Ramsden et al. (2023) also stated in their study that wheelchair basketball has positive effects on psychological health.

With the effect of the role of wheelchair basketball in increasing functionality and independence, wheelchair basketball has recently become a popular sport in which many athletes compete worldwide (Özen et al., 2016; Ramsden et al., 2023) and is becoming highly engaging and exciting for the audience (Cavalcante et al., 2022). In this respect, it is thought to be important to know the factors that motivate and maintain motivation for wheelchair basketball participation (Perreault and Vallerand, 2007).

In addition, in order to increase the participation of disabled individuals in general and wheelchair basketball in particular, it is important to understand the values and perceptions of the individual regarding the sports experience (Perreault and Vallerand, 2007; Bates et al., 2019; Ramsden et al., 2023). In this respect, there is a need for research examining the foundations of motivation from a subjective perspective (White and Duda, 1993; Perreault and Vallerand, 2007). In this context, the aim of this study is to determine the factors affecting the motivation of wheelchair basketball athletes within the framework of the self-determination theory, which was put forward by Ryan and Deci (2020) and frequently used in explaining participation motivation.

### 1.1 Theoretical framework

#### 1.1.1 Self-determination theory and motivation

Self-determination theory is one of the prominent approaches to the study of human motivation. It started in the 1970s and was developed and formulated by Edward Deci and Richard Ryan in 1980 and 1985. Since then, theory and practice have been expanded (Adams et al., 2017). According to Ryan and Deci (2017), self-determination theory deals with the social conditions that facilitate or hinder human life and has been the subject of many scientific studies. The theory examines how biological, social and cultural conditions support or undermine the individual in areas such as psychological development and health. The Self-Determination Theory is a motivational theory. In this regard, the theory utilizes motivational structures to regulate the cognitive, affective, and behavioral variables of behaviors (Deci and Ryan, 1985). This theory has also included several mini-theories, namely: organismic integration theory, cognitive evaluation theory, basic psychological needs theory, goal contents theory, causality orientations theory, and relationships motivation theory (Ryan and Deci, 2018).

According to Ryan and Deci (2017, 2020) in self-determination theory, three behavioral regulation reasons (i.e., reasons for acting) are classified as amotivation, extrinsic motivation, and intrinsic motivation. Amotivation is the absence of intrinsic and extrinsic motivation for an individual’s participation in an activity. In this respect, it falls outside motivated behaviors (Poulsen et al., 2006). Amotivation occurs as a result of not valuing an activity, feeling inadequate in doing the activity and believing that it will not yield a desired result (Ryan and Deci, 2000). The concept of amotivation is used to express how passive, ineffective, or purposeless individuals are regarding a range of potential actions, and amotivation manifests in different forms (Pelletier et al., 1999; Vansteenkiste et al., 2005). Firstly, it arises from a person’s perception that they cannot control outcomes through any action, resulting in the belief that they cannot effectively perform the required actions. A second type of amotivation does not stem from concerns about competence or control but, rather, from a lack of interest, relevance, or value. Individuals become amotivated when behaviors have no meaning or interest for them, especially when they fail to connect with the fulfillment of needs. A third type of amotivation is the apparent lack of motivation for a specific action, which is actually a motivated non-action or oppositional behavior in response to demands that thwart a basic need for autonomy or relatedness. Each of these types of amotivation may have different durations and impacts, and each has unique determinants and dynamic implications (Deci and Ryan, 2012).

Extrinsic motivation is the actions taken to gain a reward or to avoid punishment (Noels et al., 2000). Rewards, in particular, are undeniably an effective way to control behavior, but they can also reduce the development of intrinsic motivation (Ryan and Deci, 2000). Extrinsic motivation, on the other hand, requires an instrumentality between the activity and separable outcomes such as tangible or verbal rewards, so satisfaction comes not from the activity itself but rather from the extrinsic consequences to which the activity leads (Ambrose and Kulik, 1999; Gagné and Deci, 2005). For example, a student who does his homework only because his parents want him to is extrinsically motivated. Extrinsic motivation is examined in four stages: external regulation, introjected regulation, identified regulation, and integrated regulation. External regulation is carried out to satisfy or achieve an external demand for behavior. Introjected regulation is acted upon by pressures such as guilt, anxiety avoidance, and pride. Identified regulation means that the individual recognizes the personal importance of a behavior and therefore accepts self-regulation of that behavior. Integrated regulation, on the other hand, means that the individual internalizes the reasons for the action more and makes it compatible with his/her needs (Ryan and Deci, 2000).

Intrinsic motivation is an important source of energy for individuals. In the Self-Determination Theory, Ryan and Deci (2000) have defined intrinsic motivation as a prototypical expression of the active integrative tendencies in human nature. They characterize intrinsic motivation as activities done “for their own sake” or for inherent interest and enjoyment. Intrinsic motivation serves as an example of behavior that is entirely self-motivated, without any connection to external incentives or pressures, expressing solely the individual’s own satisfaction and joy (Ryan and Deci, 2020). When individuals are intrinsically motivated, they experience their interests and pleasures and feel competent and free. They internally perceive the cause of the behavior and have the opportunity to experience flow. We infer intrinsic motivation if a person performs an activity without any pressure or reward, that is, if the individual determines his/her own behavior (Deci and Ryan, 1985). It usually refers to the motivation to participate in an activity because it is enjoyable and satisfying (Noels et al., 2000), and it is seen as a psychological need (Ahmadi et al., 2023).

As mentioned above, motivation deals with the energization, regulation, and maintenance of behavior by examining why individuals act. This is particularly important in the areas of physical activity, exercise, and sports (Karataş, 2020). For example, the ability of an athlete to continue long and strenuous training sessions throughout the season with determination and motivation to participate in sports is considered to be the cornerstone of success and performance. Likewise, providing the necessary energy to participate in regular physical activity and exercise is seen as an essential element for lifelong well-being and health (Standage et al., 2019). In wheelchair basketball athletes, it is understood that the motivation that is effective in sports participation is primarily based on teamwork and the emotions derived through sports (Molik et al., 2010).

Wheelchair basketball is one of the most well-known adaptive sports, recognized not only by individuals with disabilities but also by those with typical development (Larkin et al., 2014). Wheelchair basketball players contribute significantly to the important phenomena within this sport. Understanding and interpreting the lived experiences they acquire through participation in this sport are deemed crucial. In this study, in this context, the experiences of wheelchair basketball athletes and the motivational factors within the scope of self-determination theory as a result of these experiences were tried to be understood in depth.

## 2 Method

### 2.1 Pattern of the research

This study was conducted in a phenomenological design, one of the qualitative research designs. Phenomenology investigates the lived experiences of the individual (Creswell et al., 2007) and tries to uncover the in-depth meanings of the experiences (Norlyk and Harder, 2010).

### 2.2 Study design

The study group of the research was determined using the criterion sampling method, which is one of the purposeful sampling methods. Criterion sampling can be determined in line with the criteria prepared by the criteria determined by the researchers (Patton, 2002; Yıldırım and Simsek, 2018). Accordingly, the research criteria were determined as follows.

- Participating in wheelchair basketball for at least 5 years,
- Participating in competitions in Turkey’s top league for at least 3 years,
- 22 years of age and above.

In this context, thirteen (13) athletes participating in wheelchair basketball competitions constitute the study group of the research. Information about the study group is presented in Table 1.

**Table 1:** Demographic characteristics of the study group.

Wheelchair basketball athletes							
Participant	Age	Gender	Marital status	Education status	Disability status	Year of sport	Classification (points)
P-1	35	Male	Married	High School	Amputation	12	4
P-2	48	Male	Single	High School	Paraplegia	22	1,5
P-3	29	Male	Married	High School	lymphedema	13	4
P-4	29	Male	Single	License	Amputation	12	3,5
P-5	22	Male	Single	High School	Paraplegia	5	2
P-6	27	Male	Single	High School	Paraplegia	10	2
P-7	25	Male	Single	License	Paraplegia	10	2
P-8	45	Male	Single	License	Paraplegia	23	1,5
P-9	25	Male	Single	License	Paraplegia	9	1
P-10	27	Male	Married	License	Amputation	5	3
P-11	48	Male	Married	High School	Paraplegia	22	1
P-12	25	Male	Single	Middle School	Paraplegia	5	3,5
P-13	40	Male	Married	High School	Paraplegia	13	1

When the information about the study group is examined, it is seen that the participants are between the ages of 22–48, have been practicing wheelchair basketball for 5–23 years, and train for an average of 10 h per week.

### 2.3 Data collection tool

#### 2.3.1 Personal information form and semi-structured interview form

Within the scope of the research, a personal information form was prepared to collect demographic information about the participants. With the prepared form, it was aimed to collect information such as age, educational status, disability status, number of years of participation in wheelchair basketball, classification and similar information about the participants.

Within the scope of the research, a semi-structured interview form was prepared for in-depth interviews. In the process of preparing a semi-structured interview form, a literature review was conducted. In this direction, questions were prepared by addressing the motivation phenomenon within the self-determination theory, which is one of the prominent approaches in the study of motivation and was first developed by Braun and Clarke (2017, 2020)‘s “*Self-Determination Theory’s Taxonomy of Motivation.”* Clarke and Braun (2017) noted that in studies involving thematic analysis, a deductive approach can be used for theory-driven analyses. Similarly, in our research, the interview protocol within the scope of SDT was determined through a deductive method. The prepared questions were evaluated by an academician with qualitative research competence and the questions were finalized. Some examples of questions prepared in this direction are presented below.

- Please provide information about the difficulties you encounter in your participation in wheelchair basketball. (Amotivation)
- How do the opinions of the people around you affect your participation in sports? (Extrinsic Motivation)
- In general, how do your personality traits affect your participation in sports? (Intrinsic Motivation)

In line with the prepared semi-structured interview form, the participants were interviewed face-to-face and in a quiet environment. Before the interview, all athletes were informed about the research and permission was requested for voice recording based on voluntary participation. The interviews lasted approximately 30 min for each participant. In addition, notes were taken on the issues that the participants emphasized during the interview. After the interview, the audio recordings were computerized to prevent data loss.

### 2.4 Data analyses

The reflexive thematic analysis method was used to analyze the data in the study. Reflexive thematic analysis is a frequently used method for identifying patterns of meaning in the data. It is also commonly preferred in phenomenological studies (Sundler et al., 2019). Braun and Clarke (2019) outlined the stages of reflexive thematic analysis as follows, and these stages were followed in the analysis of the data in our study.

- Analyzing the data,
- Creation of the first codes,
- Searching for themes,
- Review of themes,
- Identification and naming of themes,
- It consists of the preparation of the report.

In this context, within the scope of self-determination theory by Ryan and Deci (2000), at the end of the interview, the data were analyzed and the first codes were created. After the codes were created, themes were searched and reviewed. As a result of this process, themes were found. In this context, 6 themes and 16 sub-themes emerged. After the emergence of the themes, a metaphorical title was determined and reported for each theme.

### 2.5 Credibility, transferability, consistency and verifiability

**Figure 1:**
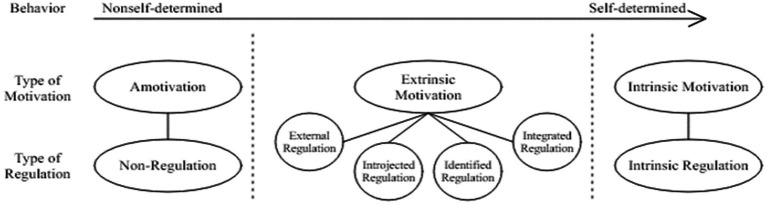
Self–determined: Adapted from Ryan and Deci (2000).

Validity and reliability in qualitative research are expressed in terms of credibility, transferability, consistency and confirmability (Lincoln and Guba, 1985; Creswell et al., 2007). In this context, the participants were informed before and after the study for the credibility and transferability. In order to ensure consistency, the research data were coded separately by an expert in qualitative research other than the researcher, and the codes were compared. In addition, quotations from the participant views were presented to the expert. Within the scope of the verifiability of the research, all transactions carried out during the research period were recorded and stored in a computerized environment (Figure 1).

## 3 Findings

**Figure 2:**
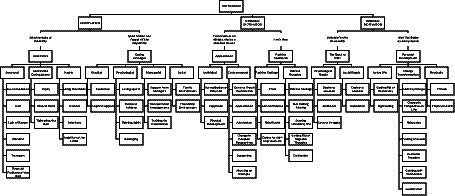
Emerging themes, sub-themes and codes.

Within the scope of the research, 6 themes and 16 sub-themes emerged. Figure 2 presents the emerging themes, sub-themes and codes.

### 3.1 Amotivation

#### 3.1.1 Disadvantage of disability: constraints

Within the theme of constraints, 3 sub-themes emerge. These sub-themes are structural, health, and constraints during sports. In this direction, it is seen that wheelchair basketball athletes have more structural limitations. Participants especially emphasized the problems of accommodation, hall, lack of ramps, materials, transportation, and financial problems of the clubs. In this context, a participant said:

“Sometimes I may have difficulties in transportation. When I come by bus and public transportation, I experience such difficulties” (P-9).

When participants are generally assessed, they indicate that one of the most significant constraints affecting participation is economic issues. It is understood that economic problems cause problems in terms of both training, equipment, salary, and travel to league competitions. One participant drew attention to the financial situation and material shortages:

“Right now it’s more economic. It’s the economy of the team, of the club. We have difficulties in such situations, whether it is the issue of chairs or away trips” (P-12).

Within the scope of health limitations, it is understood that the use of prosthetics, in addition to the existing disability, leads to new wounds and infections. In this context, the participant shares their experiences regarding the use of prosthetics as follows:

“We experience health problems the most. Regarding health, since we use prostheses, sometimes this happens in normal life, like electric shocks, tingling, pain, etc. It also happens in other parts of us, for example, many veterans have health problems such as blows to the arm, shoulder, or head during the injury” (P-1).

Another participant said:

“...Of course, there are diseases, infections, sometimes we may have problems due to certain diseases due to disabilities. We get urinary tract infections, for example, not because of sports, but because we have paraplegia, so we use a catheter. That is why we often get infections, we sometimes have problems with this” (P-11).

On the other hand, it is stated that while practicing wheelchair basketball, they experience difficulties especially due to injuries, muscle pains and tightness of the wheelchair belt. In this direction, one participant said:

“I mean, there is serious fatigue, muscle fatigue. We have muscle pains and injuries from time to time” (P-4).

The use of prosthetics due to existing disabilities in physically disabled athletes, as revealed by participant opinions, is understood to be a result of limitations in the use of body limbs, leading to challenges for athletes in this regard. On the other hand, a significant majority of the participants being paraplegic, and consequently experiencing issues related to inflection, emerges as a significant factor exacerbating existing difficulties.

#### 3.1.2 Sport makes you forget all the negativity: coping strategies

Within the scope of coping strategies, psychological, managerial, medical, and social sub-themes emerged. It is emphasized that psychological factors are more prominent in coping with the difficulties faced by wheelchair basketball athletes. In this regard, one participant said:

“The biggest thing is to love sports. You endure these constraints as you love sports. Also, setting a goal for oneself, aiming to be successful, wanting to be successful is the biggest help to cope with these difficulties, no matter which branch of sport one is in. Apart from the difficulties, you cannot cope with some constraints and you continue your sports with constraints. We have continued sports under these conditions until now...” (P-13).

On the other hand, it is emphasized that participants consider the support of team managers and coaches in every aspect as a crucial factor in overcoming challenges. A participant’s perspective on this matter is as follows.

“We are engaging when our coaches and managers provide us with these opportunities. It’s not about individual struggle; when we work alongside our coaches and managers, many problems get resolved.” (P-2).

Participants point out that one coping strategy is the creation of a social environment within the team, emphasizing its significance for them. Also, in coping with adversities, it is predominantly observed that athletes’ affection for wheelchair basketball has a significant impact. The desire to succeed against all odds is recognized as a crucial precursor in the development of this sentiment, serving as a coping mechanism against encountered constraints. In addition to these, despite facing economic challenges, the strong support from team managers and coaches, especially at the managerial level, serves as a significant motivator for athletes. Furthermore, positive and supportive interpersonal relationships within the team are also considered another factor. In this regard, the support of athletes’ families and peer groups is seen as an important strategy in overcoming encountered obstacles.

### 3.2 Extrinsic motivation

#### 3.2.1 To be seen as an athlete, not as a person with a disability: appreciation

In terms of how the participants are perceived and reacted to by the individuals around them, it is emphasized that they generally attribute meaning to the fact that they are valued. In particular, it is emphasized that the participants received positive feedback such as admiration, appreciation, and being shown as an example, which prevented their disabilities. In addition, it is underlined that there is a change in the perspectives of the individuals around them due to the fact that they are athletes. For example, one participant expressed his views on this issue as follows:

“People’s perception of me has also changed. They saw them as human beings, not as people with disabilities. They were more cordial. My friends’ thoughts changed, they saw that I was able to achieve something. They saw what a person with a disability can achieve, what I can achieve...” (P-5).

Another participant said:

“Of course, perspectives change a lot, I mean, being a national athlete is exhibited as an attitude of being pointed at in the family environment and in the environment of relatives. They say I am an athlete, they say I am in the national team. Of course, it motivates, being pointed out among people, being shown with something to be proud of has a positive effect. You get more attached to what you do, you realize that what you do is important” (P-7).

On the other hand, participants emphasize that their physical development, happiness, and the normalization process in their daily lives serve as a source of motivation. For example, a participant expresses,

“This sport keeps us in shape. My purpose for engaging in sports is entirely in this direction to be fit, that is, for the body to be fit. For instance, they say, ‘You are taking care of yourself, you are doing sports,’ because they know I engage in sports. I receive positive feedback in that regard.” (P-1).

In this context, it can be said that it is more effective for athletes to be perceived more as ‘athletes’ rather than emphasizing the obstacles. Particularly, it is emphasized that they faced more attitudes focused on hindrance in their lives before becoming athletes, but this situation changes with participation in sports. This becomes a significant source of motivation for athletes.

#### 3.2.2 I’m in now: positive feedback

It is seen that the positive feelings and thoughts arising from the fact that the individuals in the environment are athletes increase the motivation of the participants. In this context, the participants stated that the perspectives of the individuals around them created positive emotions such as pride, self-confidence, and not feeling deficient. One participant emphasized self-confidence as:

“Their opinions gave me more self-confidence. From the very beginning, I did not feel that I was lacking in any way. It increased my motivation, had a more positive effect on my performance and my sports life, and thanks to that” (P-8).

Another participant also supported these views:

“Being successful somewhere, being praised by someone else, boosts your self-confidence. This also boosts our self-esteem in a positive way. It’s like this because now I’m in it, I’m an athlete, I can achieve things too” (P-13).

Additionaly, participants frequently highlight the positive impact of individuals in their surroundings, particularly when they exhibit a positive attitude. This includes fostering positive emotions such as not feeling inadequate, overcoming negative thoughts, and generating a sense of completeness. In this regard, a participant expresses themselves as follows:

“People’s perspective has changed. They appreciated the sport I engage in, gave me extra value, and truly made me feel valuable.” (P-8).

Athletes emphasize that being seen as athletes rather than as disabled individuals and the development of positive attitudes toward them by individuals in their environment serve as a source of motivation for them in terms of building self-confidence, achieving success, and being appreciated.

### 3.3 Intrinsic motivation

#### 3.3.1 Suitable for my personality: the need to exist

The findings revealed that personality traits of wheelchair basketball athletes in general increase their motivation to participate in sports. In this direction, the fact that the participants have the desire to succeed, are ambitious, and love to struggle is the most important factor in practicing this sport. For example, one participant expresses himself in this regard as follows:

“I am ambitious, I cannot tolerate losing” (P-7).

In this direction, another participant expressed her feelings as follows:

“Basketball is a sport that fits my personality. It’s a challenging sport, that’s how it is in life. I want to take part in a sport where there is extreme struggle, where there is a real war inside, plus the ambition to win, the desire to win, this is also in people’s daily lives. The same is true in basketball” (P-8).

Another factor is that participants emphasize the significance of wheelchair sports as a motivation for their desire to socialize and express their emotions. A participant shares,

“Sometimes, when people mention it, they laugh. Before getting into sports, before the injury, my circle of friends was so rare. Seeking dialog with people, even trying to integrate into the environment, was a challenging task. However, this situation turned out to be a blessing. Thanks to the team, thanks to the community, my circle of friends has expanded so much that sometimes I think to myself, ‘I’m glad I faced this constraint, I’m glad I reached this point.’ Not only has my circle of friends expanded, but also my social activities have increased. When people say, ‘Let us go, let us do it,’ this time, people include me in their plans. It’s a benefit of sports.” (P-10).

It is emphasized that the most crucial factor in ensuring intrinsic motivation is psychological factors. Particularly, in the development of this condition, it is understood that athletes’ general personality traits, such as being resilient, ambitious, and intolerant of losing, play an effective role. In addition, it is observed that through sports, they spend more time in social environments and engage in settings that provide participants with the opportunity to express themselves better.

#### 3.3.2 We will get better by doing sports: personal development

It is seen that there are many positive changes in the lives of the participants in wheelchair sports and these changes increase their motivation to participate in sports. Especially in this regard, it is emphasized that identity change occurs and that they gain self-confidence. Participants also reported that their perspectives on life changed, a social environment was provided, and there was a physical change and transformation. Participant views on this issue are given below:

“It gives you an athlete identity, if nothing else, it gives you an athlete identity. Athlete identity is not something that everyone can easily do nowadays” (P-1).

“Sports gave me self-confidence, it gave me the ability to fight with life. I actually learned that I can overcome difficulties. I mean, it activated me, quite frankly, against life. Sports has given me a lot. Power, thought, freedom, in other words, activism, that is, a lot of things related to life” (P-12).

“I am an active person. I enjoy having fun and entertainment. I prefer group settings more and do not like being alone. I can say that wheelchair basketball has been quite supportive for me in this regard.” (P-6).

In this context, within the framework of participant opinions, it is observed that the emphasis is on engaging in wheelchair basketball to highlight the prominence of personal development through sports and the idea that we will get better by participating in sports.

## 4 Discussion

This study was conducted with the aim of determining the amotivation of wheelchair basketball players in their participation in sports, the factors affecting their extrinsic motivation, and how they provide intrinsic motivation within the scope of the self-determination theory put forward by Ryan and Deci (2000). The findings obtained in this context are discussed and interpreted below.

### 4.1 Amotivation

#### 4.1.1 Disadvantage of disability: constraints

In the context of the findings, it is understood that wheelchair basketball athletes face many constraints. In a study conducted by Bentzen and Malmquist (2022), which employed the same theory as our research, the satisfaction of needs in physical education, organized sports, and self-organized physical activity among adolescents with disabilities compared to their peers without disabilities over a three-year period was examined within the framework of SDT. The study concluded that adolescents with disabilities have lower participation, especially in physical education and partially in organized sports activities. In this context, it was emphasized that the suitability of structure is crucial for reducing constraints. In this regard, Argan et al. (2021) concluded in their research that there are structural barriers especially due to transportation and access. The research emphasizes that there are significant difficulties, from transportation to facilities to accessibility to halls and halls and lack of ramps. Similarly, Jaarsma et al. (2014) state that wheelchair athletes have difficulties accessing facilities and materials compared to other physically disabled athletes. On the other hand, it is also seen that athletes have health problems due to disability and difficulties they experience while playing basketball. Esatbeyoğlu and Karahan (2014) state that especially people with physical disabilities face more constraints in participating in sports than those with hearing and visual impairments. When the literature on this subject is examined, it is seen that the problems of wheelchair basketball players are divided into diseases that are not related to basketball sports and injuries experienced while playing basketball (Sá et al., 2022). As a reason, it is stated that the pain in the spine area, especially in the lower back, is high as a result of overload, especially in those who use wheelchairs continuously (Curtis and Black, 1999; Wilroy and Hibberd, 2018; Sá et al., 2022). In the scope of our research, it is understood that there are more limitations related to structural factors based on the acquired data. Primarily, the construction of a structure specific to the current disability conditions of disabled athletes is emphasized. It is believed that the facilitation of individuals’ inclusion in activities will be enhanced within this facilitative context. Additionally, it is considered crucial to take precautions to prevent the occurrence of secondary disabilities alongside their existing disability conditions. The findings suggest that the disability level varies for each disabled athlete in the teams, and their changing needs in this context should be taken into account.

#### 4.1.2 Sport makes you forget all the negativity: coping strategies

It is seen that psychological factors are especially at the forefront in coping with these negativities faced by wheelchair basketball athletes (Perreault and Vallerand, 2007; Martin et al., 2015). It can be said that performing the best performance under pressure, concentration, self-confidence, the ability to set goals for oneself, as well as believing that one can be trained and having a positive relationship with oneself are effective in coping with difficulties (Perreault and Vallerand, 2007). In this regard, Lundberg (2006) examined the sports participation and subjective well-being of individuals with spinal cord injuries within the scope of SDT. The research concluded that providing additional sports opportunities for individuals with spinal cord injuries, especially in the post-treatment period, is effective in enhancing their psychological strength. Also, Erdemir et al. (2009) state that physical disabilities do not constitute a constraint to playing basketball; on the contrary, they are important in terms of showing that they are a part of society and that they see basketball as a joy of life. In this context, it can be said that sports make people forget the difficulties and troubles they experience. Within the scope of the obtained findings, it is possible to discuss some psychological issues caused by the existing constraints of wheelchair basketball players. However, it is observed that individuals create an outlet for themselves in terms of factors such as performing well in sports, the desire for success, and concentration. In this context, it can be said that individuals are ‘better’ both physically and psychologically. Therefore, basketball has been adopted as an important ‘coping mechanism’ for wheelchair basketball players.

### 4.2 Extrinsic motivation

#### 4.2.1 To be seen as an athlete, not as a disabled person: appreciation

According to the findings of the study, it was determined that wheelchair basketball athletes were appreciated, shown as an example, and looked at with admiration by the individuals around them. Perreault and Vallerand (2007) define extrinsic factors as a significant variable within the scope of SDT when individuals participating in adapted sports explain their involvement in their respective sport. In this direction, they see being seen as an athlete, not as a person with a disability, as an important source of extrinsic motivation for them. Kumcağız and Çayir (2018) emphasize the importance of family members, friend groups, coaches and state support. They reported that this motivated them and helped them to cope with difficulties. In this regard, Ramsden et al. (2023) underlines that being in a sports environment changes attitudes toward people with disabilities. It is reported that not only intrinsic motivations are not sufficient for the participation and encouragement of individuals with disabilities in sports, but also extrinsic reasons should be taken into account (Perreault and Vallerand, 2007). In this direction, it can be said that the fact that wheelchair basketball athletes are valued by the individuals around them is an important motivational factor for them to be seen as an athlete, not as a person with a disability. The obtained findings indicate that wheelchair basketball serves as a means for disabled athletes to be appreciated and, in fact, accepted within society. In reality, extrinsic factors seem to be crucial for disabled athletes, and the opinions of external factors (*family, friends, neighbors,* etc.) impact and add meaning to their lives.

#### 4.2.2 I’m in now: positive feedback

It is seen that these positive attitudes of the individuals in the environment toward wheelchair basketball athletes positively affected the participants. In this regard, in studies conducted in a similar field, SDT-based physical activities were seen to enhance individuals’ participation in physical activity, their quality of life, and their levels of happiness (Ghaneapur et al., 2019). Accordingly, McLoughlin et al. (2017) concluded that the social support perceived by athletes with physical disabilities is effective in their motivation to participate in sports. Again, it has been determined that the social support received by athletes with physical disabilities from the individuals around them positively affects participation in sports (Wessel et al., 2011). Jeffress and Brown (2017) stated in their study that individuals with physical disabilities are accepted and respected by society regardless of their physical abilities. It is reported that this situation leads to an increase in the self-efficacy beliefs of the participants, thus enabling an increase in the quality of life of individuals with physical disabilities. In this context, it can be said that the positive social support that individuals with disabilities receive directly or indirectly is important in their motivation to participate in a physical activity (Durmuş et al., 2021; Olsen et al., 2023). Within the scope of the research findings, it can be stated that increasing encouraging factors for the participation of disabled athletes in sports and promoting their participation could enhance their respective motivations. This situation is thought to be achievable by increasing the visibility and awareness of wheelchair basketball and other disabled sports, both as a sport and its athletes, in society. It can be said that any form of positive feedback serves as a significant motivational source for disabled athletes, empowering them through such encouragement.

### 4.3 Intrinsic motivation

#### 4.3.1 Suitable for my personality: the need to exist

According to the research findings, it is concluded that the intrinsic motivation of wheelchair basketball athletes affects their participation and enthusiasm in sports. Accordingly, Banack et al. (2011) examined the relationship between Paralympic athletes’ perceptions of autonomy-supportive coach behavior, basic psychological needs, and intrinsic motivation to know, accomplish, and experience stimulation within the framework of SDT. The study emphasizes the significance of the alignment of factors influencing athletes’ intrinsic motivation. It can be said that, through this alignment, athletes with disabilities can be successful. Also, it is seen that the fact that they have an ambitious and combative structure is a factor in this situation. Sports is seen as one of the most effective factors that increase the fighting spirit of individuals with disabilities to survive and hold on to life despite all the difficulties of life (Soyer et al., 2013; Gürkan et al., 2021). The fact that athletes with physical disabilities love competition and have the desire to achieve goals positively affects their motivation to participate in sports (McLoughlin et al., 2017). It is stated that the motivation of individuals with physical disabilities to participate in physical activity is influenced by the desire to achieve performance, the desire to succeed, and the spirit of competition (Mindrescu, 2022). In this direction, it can be concluded that the participants have a personality trait compatible with wheelchair basketball sport. According to the obtained data, it can be said that the desire for success and the combination of competitive elements increase the intrinsic motivation of wheelchair basketball players. In this context, it is considered important for them to choose a sports branch that suits their personalities, allowing them to express themselves in society. Additionally, it can be stated that the participants’ choice of wheelchair basketball is influenced by their ambitious and competitive identities.

#### 4.3.2 We will get better by doing sports: personal development

Participants stated that experiencing positive changes in their participation and continuation in sports supported them internally. For example, Çil et al. (2023) stated in their research that individuals with physical disabilities who do sports have an increase in positive thinking skills compared to those who do not do sports. Likewise, in their study on wheelchair basketball athletes, Fiorilli et al. (2013) stated that individuals who participate in basketball sports have more psychological well-being and social skills than those who do not. Ramsden et al. (2023) state that the establishment of friendships and belonging to a community are important for participation in wheelchair basketball. Again, in a study conducted by Tekkurşun-Demir and İlhan (2020), it was concluded that athletes with physical disabilities minimize their sports inadequacies and encounter more limitations than other disabilities. In our research, it can be said that wheelchair basketball players not only engage in sports but also make an effort to find the meaning of life through this branch. Through participation in sports, individuals are seen to push their existing potentials to the highest level. It can be stated that individuals also improve their quality of life through participation in sports. This situation is considered to be effective in athletes having a more optimistic/positive outlook on the future.

## 5 Conclusion

The aim of this study is to determine the motivational factors in the participation of wheelchair basketball players in sports according to the Self-Determination Theory. Within the scope of the findings obtained, it can be concluded that the most significant constraints for wheelchair basketball players arise from structural limitations such as financial difficulties, equipment, transportation, and accommodation issues faced by clubs. On the other hand, the use of prosthetics, infectious diseases related to disability, and difficulties in using limbs can be considered as disadvantages of the disability. Wheelchair basketball players’ desire to overcome these challenges and constraints, along with psychological factors such as the will to succeed and a fighting spirit, together with managerial support and the club environment being a family atmosphere, are identified as the most important coping strategies.

Another result obtained in the study is that wheelchair basketball players are externally motivated more by positive attitudes and behaviors from their surroundings. In the formation of this situation, the recognition, exemplification, and support of athletes by individuals in their environment, such as family, friends, and neighbors, play a crucial role. Internally motivating factors are primarily driven by the athletes’ existential needs and the desire for self-improvement. In this regard, the most important factors contributing to these aspects are identity change, a shift in perspectives on life, and an increase in quality of life.

## 6 Strengths and limitations

This research’s strengths lie in examining the factors influencing wheelchair basketball players in overcoming obstacles and using qualitative research methods within the framework of the Self-Determination Theory (SDT). In this regard, it aims to provide detailed and in-depth information that will enable a comprehensive understanding of the behaviors of wheelchair basketball players in overcoming obstacles and the processes of change in their sports lives, within their own context. It is also believed that the study can contribute significantly to the relevant literature with the existing information. The limitations of the research include the difficulty in generalizing the results, as the data were collected from a limited number of participants.

## Data availability statement

The original contributions presented in the study are included in the article/supplementary material, further inquiries can be directed to the corresponding author.

## Ethics statement

Ethical review and approval was not required for the study on human participants in accordance with the local legislation and institutional requirements. The participants provided their written informed consent to participate in this study.

## Author contributions

HS: Conceptualization, Data curation, Formal analysis, Funding acquisition, Investigation, Methodology, Project administration, Resources, Software, Supervision, Validation, Visualization, Writing – original draft, Writing – review & editing.
